# Flow visualisation of a normal shock impinging over a rounded contour bump in a Mach 1.3 free-stream

**DOI:** 10.1007/s12650-016-0392-4

**Published:** 2016-09-02

**Authors:** Kin Hing Lo, Konstantinos Kontis

**Affiliations:** University of Glasgow, University Avenue, Glasgow, G12 8QQ UK

**Keywords:** Rounded contour bump, Normal shock structure, Shock generator, Lambda shock structure, Supersonic wind tunnel

## Abstract

**Abstract:**

An experimental study has been conducted to visualise the instantaneous streamwise and spanwise flow patterns of a normal shock wave impinging over a rounded contour bump in a Mach 1.3 free-stream. A quartz-made transparent shock generator was used, so that instantaneous images could be captured during the oil-flow visualisation experiments. Fluorescent oil with three different colours was used in the surface oil-flow visualisation experiment to enhance the visualisation of flow mixing and complicated flow features that present in the flow field. Experimental data showed that the rounded contour bump could split the impinging normal shock wave into a or a series of lambda-shaped shock wave structure(s). In addition, it was found that the flow pattern and the shock wave structures that appeared over the rounded contour bump depended highly on the impinging location of the normal shock wave. The flow pattern shown in this study agreed with the findings documented in literature. Moreover, it was observed from the instantaneous oil streaks that the normal shock impinging location also affected the size and the formation location of the spanwise counter-rotating vortices downstream of the bump crest. Finally, it was concluded that the terminating shock could distort the oil streaks that left over the surface of the contour bump. Therefore, the use of the transparent normal shock wave generator is recommended when conducting experiments with normal shock wave impingement involved.

**Graphical abstract:**

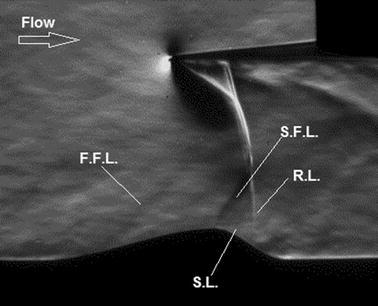

## Introduction

Drag reduction on transonic aircraft wings using contour bumps or shock control bumps has been an active and ongoing research topic in both the academia and industry. In fact, the effects of two- and three-dimensional contour bumps in achieving drag reduction on aerofoils and finite wings have been well documented in literature (e.g. Rosemann et al. 2003; König et al. 2007, 2009; Qin et al. 2004, 2008; Wong et al. 2008; Birkemeyer et al. 2000; Pätzold et al. 2006; Kutzbach et al. 2004, etc.). In general, about 5–15 % of drag reduction could be achieved with an aerofoil or finite wing equipped with contour bumps compared with the baseline aerofoil/finite wing geometry. If contour bump geometry is optimised, the numerical simulation results documented in Kutzbach et al. (2004), and Milholen and Owen (2005) showed that about 14–15 % of drag reduction could be achieved by installing an optimised two-dimensional contour bump on an aerofoil. The numerical simulation results shown in Qin et al. (2008) concluded that 18.2 and 20.1 % of drag reduction could be achieved on a RAE5243 aerofoil equipped with optimised two- and three-dimensional contour bumps, respectively. It is interesting to note here that the analytical study conducted by Ogawa and Babinsky (2006) showed that a potentially 85 % of wave-drag reduction could be achieved when implementing an ideal two-dimensional contour bump into a transonic aerofoil. Recently, Lee et al. (2013) using numerical simulations showed that 80 % of wave-drag reduction is possible to be achieved by an aerofoil equipped with an optimised two-dimensional contour bump in transonic free-stream. Although the previous studies showed the promising results in achieving drag reduction on transonic aerofoils/wings equipped with contour bumps, there is a major issue associated with using contour bumps in attempt to achieve drag reduction. In fact, contour bumps only provide desirable effects during designated operational conditions. König et al. (2009) reported that drag enhancement could be occurred in a contour bumps equipped aerofoil during off-design operations due to the presence of unfavourable flow features over the bump surface. Therefore, it is necessary to understand the flow physics of contour bumps before they can be successfully applied to achieve drag reduction in transonic aircraft.

The flow physics of contour bumps in transonic/supersonic free-stream has been extensively studied in recent years. A review article written by Bruce and Colliss (2015) in this subject area was recently published. The operational principle of a shock control bump in achieving wave-drag reduction is by spitting a normal shock wave that impinges over the surface of an aircraft wings into a or a series of weaker lambda shock wave structures (Ashill et al. 1992; Fulker et al. 1993). Ideally, the contour bump should be situated in a position, so that the normal shock is impinging over the bump crest region to prevent the formation of the adverse flow features downstream of the shock impingement location (Babinsky and Ogawa 2007; Bruce and Babinsky 2012; Bruce and Colliss 2015). However, the location of normal shock impingement over the surface of the aircraft wings varies continuously in different flight regimes. Therefore, it is difficult to ensure that the normal shock wave could always impinge over the bump crest in practical situations. Although the concept of “adaptive bumps” was proposed by Birkemeyer et al. (2000), these shock control bumps are driven by heavy mechanical actuators that make them become impractical to be used on actual aircraft.

The problem of normal shock wave impinging over the surface of various shock control bumps has extensively been investigated by Ogawa et al. (2008), Bruce and Babinsky (2012) and Colliss et al. (2014). The authors in these studies concluded that unfavourable flow features like second lambda shock wave and expansion waves could appear over the surface of the shock control bumps when the normal shock is impinging ahead or behind the bump crest region. The presence of these unfavourable flow features leads to drag enhancement. One way to reduce the adverse effects induced by the wedge-shaped shock control bumps during off-design conditions is to increase the length and reduce the crest height of the bumps (Ogawa et al. 2008; Bruce and Babinsky 2012; Bruce and Colliss 2015). Instead of using wedge-shaped bumps, König et al. (2007, 2009) studied, both experimentally and numerically, the effectiveness of using three-dimensional rounded contour bumps in achieving drag reduction on transonic aerofoils. The author concluded that rounded contour bumps are more effective than wedge-shaped shock control bumps in achieving wave-drag reduction on transonic aircraft wings. Although the authors did not study the flow physics of rounded contour bumps, the authors observed that a large pair of spanwise, counter-rotating vortices was formed downstream of the bump crest that could significantly increase the pressure drag encountered. The authors deduced that the formation of this spanwise vortex pair was caused by the flow separation that appears immediately downstream of the bump crest. In fact, similar spanwise vortical structures were also documented in Yang et al. (2012a), Tao et al. (2013) and Bruce and Colliss (2015). Recently, Lo (2014), Lo and Kontis (2015, 2016), and Lo et al. (2013, 2015, 2016) experimentally studied the flow pattern over a rounded contour bump without normal shock wave impingement in both Mach 1.3 and 1.9 free-stream. The authors in these studies showed that a large pair of counter-rotating spanwise vortices was formed downstream of the bump crest. In addition, some other interesting flow features, such as the separation shock, re-attachment shock, expansion waves, and shear layer, could be observed over the surface of the rounded contour bump model.

Although the flow physics of shock control bumps has extensively been studied experimentally in the last two decades, only non-instantaneous flow pattern was shown in most of these studies. This is because the presence of the opaque normal shock wave generator usually prevents optical access from the top of the wind tunnel test section. As a result, some flow diagnostic techniques, such as particle image velocimetry (PIV), particle shadow velocimetry (PSV), and instantaneous surface oil-flow visualisation, etc., become difficult to be implemented. In addition, Bruce and Babinsky (2012) mentioned that the oil streaks left over the model surface could be distorted by the backward moving terminating shock that appears during the wind tunnel stopping period. To be exact, the spanwise flow pattern captured during the wind-off condition after the wind tunnel test could be deviated from the wind-on flow pattern. Recent developments in silicon glass product machining and addictive manufacturing techniques suggest that normal shock wave generators could become transparent. This means that some experimental techniques that could not be or difficult to be implemented previously due to the opaque nature of the conventional normal shock wave generators could now be adopted to resolve the instantaneous flow pattern over the surface of the contour bump models.

The purpose of this study is to re-investigate the problem of normal shock wave impinging over a three-dimensional rounded contour bump in a Mach 1.3 free-stream using two novel experimental strategies. First, a quartz-made transparent normal shock wave generator was used to generate the impinging normal shock, so that instantaneous streamwise and spanwise flow pattern over the contour bump could be visualised. The advantages and potential shortfalls of using this transparent shock wave generator are discussed. In addition, a novel surface oil-flow visualisation technique which uses fluorescent dye with multiple colours is introduced. It is believed that using multiple colour dye could enhance the visualisation of flow mixing and other complicated flow features that present in the flow field. In addition, the spanwise flow pattern captured during wind-on and wind-off conditions is compared with justify the value of using transparent shock generator in experiments that involve normal shock wave impingement. Finally, the results obtained from this study could review the flow physics over a rounded contour bump in supersonic free-stream. Since this study aims to introduce the use of the transparent shock wave generator and the multiple colour surface oil-flow visualisation technique, therefore, only high-speed monochrome Schlieren photography and surface oil-flow visualisation were used for flow diagnostics. It is believed that the data collected from this study could indicate the importance of capturing instantaneous flow pattern during supersonic wind tunnel tests.

## Experimental setup and flow diagnostics

### Transonic/supersonic wind tunnel

Experiments were conducted using an intermittent in-draft transonic/supersonic wind tunnel which means that the flow is generated and maintained by a pressure difference between the atmosphere and vacuum. The same wind tunnel was previously employed in a wide range of transonic and supersonic aerodynamic related studies. The details of this wind tunnel can be found in Lo (2014), Lo and Kontis (2015, 2016), Lo et al. (2013, 2015, 2016), Zare-behtash et al. (2014, 2015, 2016), and Ukai et al. (2014a, b). Therefore, only a brief description on this wind tunnel facility is provided here.

The wind tunnel has a rectangular test section and it has the dimensions of 485.5 mm (length) × 150 mm (width) × 210 mm (height). A quick opening butterfly valve is situated between the test section and the vacuum tank, so that a pressure difference is developed when the valve is opened. A pair of convergent-divergent nozzles situated upstream of the test section is used to generate the required Mach 1.3 supersonic free-stream. The free-stream Mach number was calculated using the total pressure ratio between two points with one point located at the settling chamber, while another one located at the test section. Pitot probes were used for the pressure measurements in calculating the free-stream Mach number. The rear end of the pitot probes was connected to two Kulite XT-190 M pressure transducers via flexible tubes. Voltage signals generated by the pressure transducers were captured by a National Instruments (NI) NI USB-6259 Data Acquisition (DAQ) System at a sampling rate of 20 kHz over a sampling period of 10 s. The flow Reynolds number per unit length (*Re*/*L*) is *Re*/*L* = 12.11 × 10^6^. Optical access to the wind tunnel is achieved through two side windows and a ceiling mounted window. All of these windows are made of quartz. The wind tunnel has a stable runtime of 6 s (Lo and Kontis 2016; Lo et al. 2013, 2015, 2016; Zare-Behtash et al. 2014, 2015, 2016; Ukai et al. 2014a, b). Under the same initial conditions, the variation of the free-stream Mach number (*M*_*∞*_) is about *M*_*∞*_ = 1.3 ± 0.1.

### The rounded contour bump and normal shock generator

**Fig. 1:**
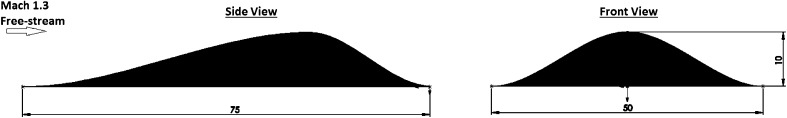
Schematic diagram of the three-dimensional shock control bump model

A three-dimensional rounded contour bump model with geometry shown in Fig. 1 was used. The model was made of Grade 6083 aluminium alloy. It has the dimensions of 75 mm (length) × 50 mm (width) × 10 mm (crest height). The same bump geometry was also employed in the numerical studies about drag reduction in transonic aircraft wings by Qin et al. (2004, 2008) and Wong et al. (2008) as well as the experimental studies about the flow physics of contour bump by Lo (2014), Lo and Kontis (2015), Lo et al. (2013, 2015, 2016) and recently Zare-Behtash et al. (2016). The contour bump model was floor mounted at 82.5 mm downstream of the front along the centreline of the wind tunnel test section.

**Fig. 2:**
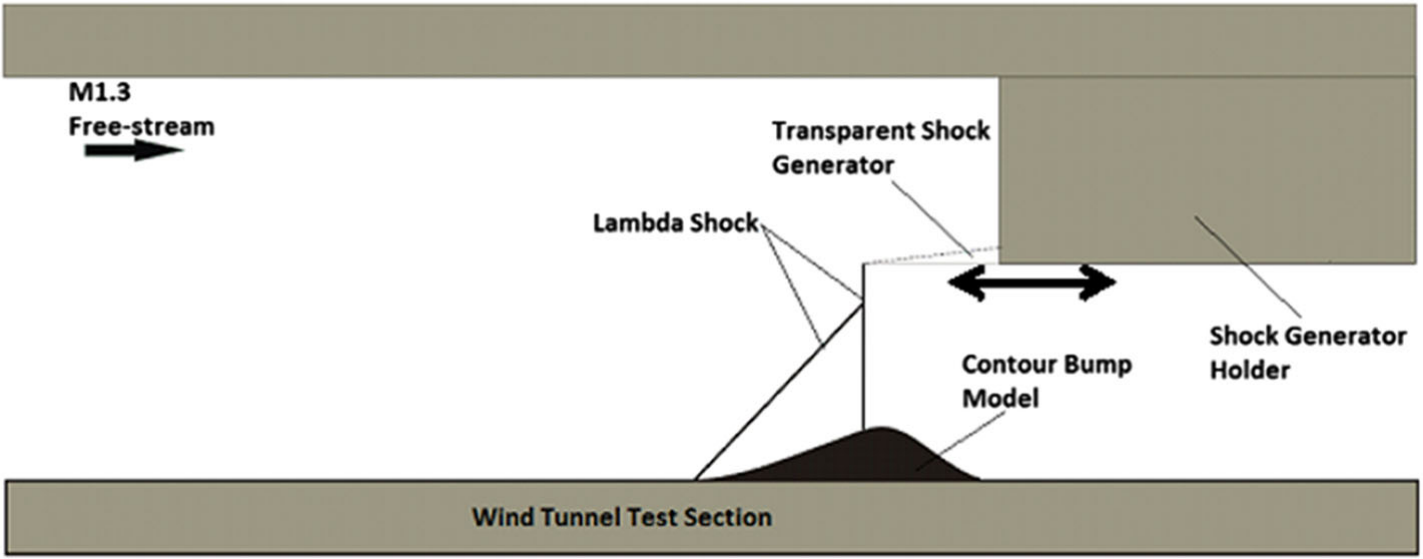
Schematic setup of the experiment

The normal shock wave that used to impinge over the surface of the contour bump model was generated by a quartz-made transparent normal shock wave generator. This normal shock generator has the dimensions of 105 mm (length) × 144 mm (width) × 6 mm (thickness). It was suspended from the ceiling of the wind tunnel test section, so that a height of 80 mm between the floor and the normal shock generator is maintained. It can move along the axial direction, so that the nearly normal shock wave generated can be impinged over the surface of the rounded contour bump model at various streamwise locations. As already mentioned, the transparent nature of quartz means that instantaneous oil streaks could be captured during the surface oil-flow visualisation experiments. This is particularly important, as a similar study conducted by Bruce and Babinsky (2012) using wedge-shaped bumps shows that the oil streaks captured during the wind-off condition after the wind tunnel operation could be distorted by the moving terminating shock wave. The schematic setup of the experiment is shown in Fig. 2. The normal shock wave generated was impinged at various normalised streamwise locations (*l*/*c*) over the surface of the rounded contour bump. Totally six normalised locations ranging from *l*/*c* = 0.125–1 were considered. It should be noted that *l* and *c* are the local position in the *x*-direction and the total length of the rounded contour bump model (i.e. *c* = 75 mm), respectively.

### Monochrome high-speed schlieren photography technique

The streamwise flow pattern over the rounded contour bump model was examined using the conventional monochrome high-speed Schlieren photography technique. A Topler’s z-type Schlieren photography setup was employed. It should be noted that the same setup was also employed by Yang et al. (2012b), Lo (2014), Lo and Kontis (2015), Lo et al. (2013, 2015, 2016), and Ukai et al. (2014a, b). Illumination was provided by a 450 W arc-lamp light source. The light beam was first focused by a focusing lens and passed through a 2 mm-wide slit situated at the focal point of the light beam. The focused light beam was then expanded and reflected by the first parabolic mirror that has the diameter and focal length of 203 and 2088 mm, respectively. The reflected light beam illuminated the wind tunnel test section and then reflected again by the second parabolic mirror that has the same size and focal length as with the first parabolic mirror. The light signal reflected from the second parabolic mirror was first passed through a horizontal knife edge, so that the sensitivity of the Schlieren images could be adjusted. A set of Hoya 49 mm close-up lens was placed behind the knife edge and the light signal transmitted was capture by a Photron FastCam SA.1.1 high-speed camera. The frame rate, resolution, and the exposure time of the camera were set to 5000 frames per second, 1024 × 1024 pixels, and 1 µs, respectively.

### Surface oil-flow visualisation

The spanwise flow pattern over the contour bump model was investigated using the surface oil-flow visualisation technique. It should be noted that similar experimental setup was also used in Lo (2014), Lo et al. (2013, 2015, 2016) and recently Zare-Behtash et al. (2016). Fluorescent dye with three different colours (i.e., green, red, and light blue) was prepared and used in this study. It is believed that the use of multiple colour dye could enhance the visualisation of the complicated flow features and flow mixing in the flow field. The fluorescent oil was applied 100 mm upstream of the contour bump model by pipettes. Illumination was achieved through a pair of ultra-violet (UV) light-emitting diode (LED) panels with the peak emission spectrum of 395 nm. These UV LED panels were placed at the two sides of the wind tunnel test section, so that the entire test section was uniformly illuminated. To improve contrast, the contour bump model was painted with five layers of matt black paint prior to the experiments. During the experiments, the instantaneous oil streaks left on the model surface were captured by a ceiling mounted Cannon EOS 600D Single Lens Reflection (SLR) camera that has a maximum resolution of 14 mega-pixels. The ISO speed, aperture size, and shutter speed of the camera were set to ISO400, F11, and 1/4000 s, respectively.

## Results and discussion

### Flow pattern over the contour bump

**Fig. 3:**
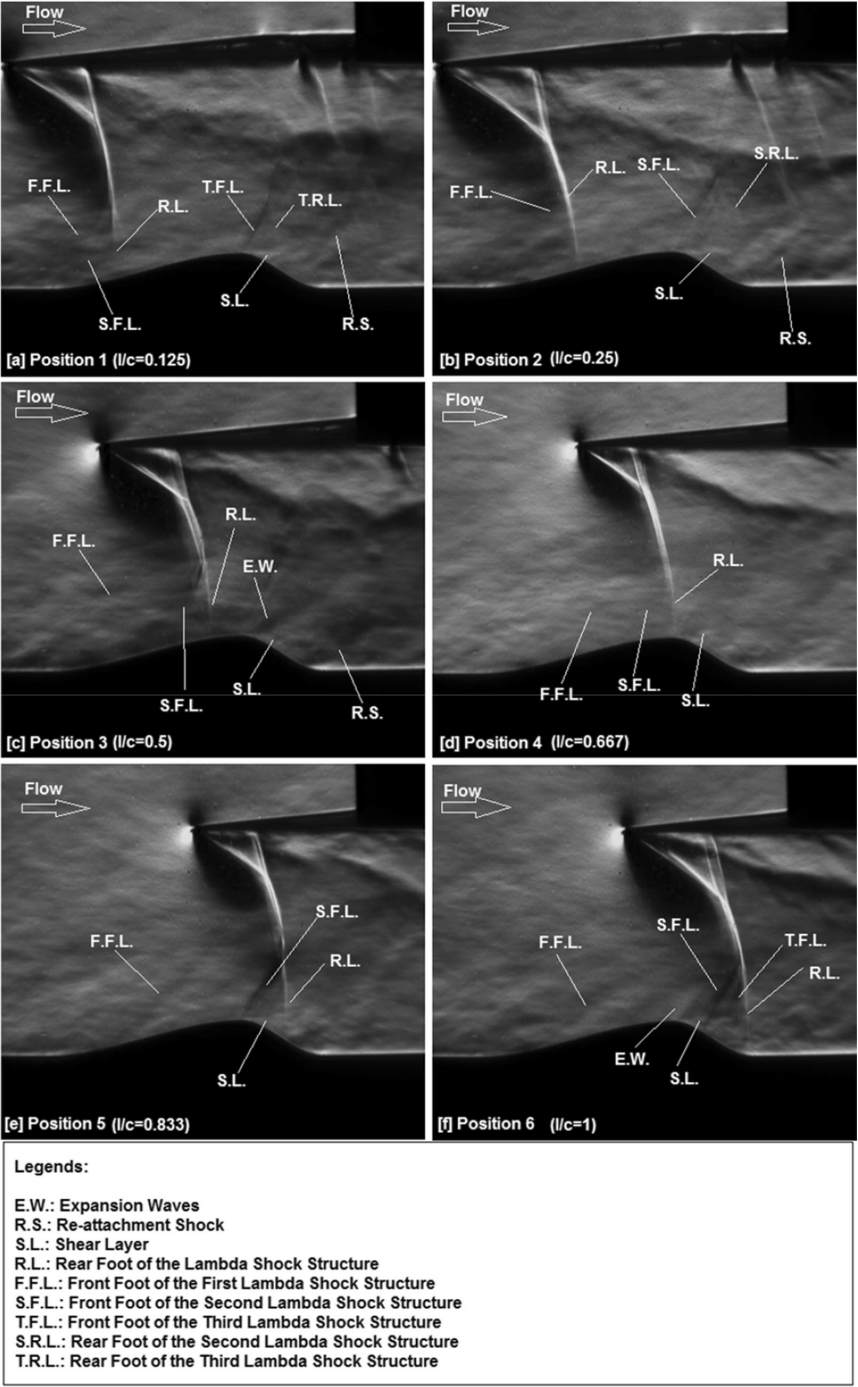
Schlieren photography images of the nearly normal shock impinging over the surface of the rounded contour bump at various normalised (*l*/*c*) locations. **a** *l*/*c* = 0.125, **b** *l*/*c* = 0.25, **c** *l*/*c* = 0.5, **d** *l*/*c* = 0.667 (i.e., the bump crest), **e** *l*/*c* = 0.833, and **f** *l*/*c* = 1 (i.e., the rear end of the contour bump)

The streamwise flow pattern along the rounded contour bump model with a normal shock impinging over its surface at various normalised locations is first shown in Fig. 3 in the form of Schlieren photography images.

From Fig. 3a, b, it can be seen that the normal shock generator is tilted slightly downwards. This is due to the fact that the quartz-made shock generator is considerably heavy and the design of the wind tunnel test section limits the number of anchoring points that could be provided to support the shock generator. In addition, because of the brittle nature of quartz, a small radius is required to be provided at the leading edge of the normal shock generator. Resulting from these two shortfalls, the normal shock that generated by the shock generator is slightly curved. Although the normal shock wave generated was not perfectly straight, as would be shown later in this subsection that the flow features that appear over the bump model is comparable with those documented in literature, such as Bruce and Babinsky (2012) and Bruce and Colliss (2015). In fact, a closer examination of Fig. 3 shows that the portion of the normal shock wave adjacent to the bump surface is straight. Therefore, it is believed that the experimental data collected should not be significantly compromised by the slightly curved normal shock that was generated. Moreover, as the aim of this study is to introduce the transparent normal shock generator that could be used to obtain instantaneous flow pattern in experiments involved normal shock impingement. With the state-of-the-art addictive manufacturing technology, it is feasible to manufacture much lighter, sharper, and stiffer transparent normal shock generators using polymer-based materials.

After describing one of the potential shortfalls in using the quartz-made transparent normal shock generator, the flow pattern over the rounded contour bump is investigated. When the normal shock is impinging at the normalised location *l*/*c* = 0.125 (i.e., position 1, Fig. 3a) over the surface of the contour bump, at the front of the contour bump, the impinging normal shock is split into a lambda-shaped shock wave structure with at least two front shock segments (F.F.L and S.F.L) and one rear nearly normal shock segment (R.L.). From Fig. 3a, it is clear that these front legs are weak, as they could only be barely seen from the Schlieren photography image. The presence of the considerably weak oblique shock/compression waves at the front of the rounded contour bump is because of the three-dimensional flow relieving effects as concluded by König et al. (2009). This is contradictory to the Schlieren images shown in Bruce and Babinsky (2012), where considerably strong oblique shock segment was formed at the front of a wedge-shaped bump in a Mach 1.3 free-stream. It is generally believed that higher pressure recovery could be achieved in rounded contour bumps over wedge-shaped bumps due to the presence of weaker lambda shock wave structures. Downstream of the rear leg (R.L.) of the first lambda shock, the flow, re-expands and accelerates to become supersonic along the ramp-shaped front surface of the bump. At the bump crest, a second lambda shock structure is formed and this lambda shock structure has a clearly defined front oblique shock/compression wave segment (T.F.L.) and a weak rear normal shock segment (T.R.L.). It should be noted that similar second lambda shock structures were also reported by Bruce and Babinsky (2012) and Ogawa et al. (2008). The presence of this second lambda shock structure is unfavourable. It increases the wave drag encountered by the bump as well as leads to the occurrence of flow separation downstream of the rear leg of the second lambda shock structure (T.R.L.) at the bump crest. This is evidenced by the presence of the shear layer (S.L.) at the bump crest which indicates that flow separation begins there. Similar flow separation phenomenon at the bump crest was also documented in Bruce and Babinsky (2012) and Ogawa et al. (2008) using wedge-shaped bumps.

Figure 3b shows the flow pattern of the rounded contour bump when the impingement location of the normal shock wave is shifted to a slightly downstream location (i.e., *l*/*c* = 0.25) along the bump front surface. In general, the flow pattern remains similar to that shown in Fig. 3a when the normal shock impingement location is at *l*/*c* = 0.125 although the first lambda shock structure (F.F.L. and R.L.) becomes bigger in size. At the bump crest, a second lambda shock structure (S.F.L. and S.R.L.) could be barely seen in Fig. 3b. This indicated that the second lambda shock structure that formed is considerably weak as less distance is available behind the rear leg of the first lambda shock for the flow to re-accelerate before reaching the bump crest. To be exact, the local flow speed at the bump crest is lowered in the present case, where the shock impingement location is at *l*/*c* = 0.25, and therefore, weaker second lambda shock structure is formed consequently. Flow separation could be observed clearly behind the rear leg of the second lambda shock (S.R.L.), as shown in Fig. 3b. As a result, a clearly defined shear layer (S.L.) is present at the bump crest.

When the shock impingement location is moved further downstream to *l*/*c* = 0.5 (Fig. 3c), the first lambda shock structure, featured with at least two front oblique shock segments (F.F.L. and S.F.L.) and a rear normal shock segment (R.L.), becomes larger and more clearly defined. Since the rear leg of the lambda shock structure (R.L.) is located further downstream in the present case, flow re-expansion is limited to a very short distance before reaching the bump crest. As a result, instead of forming the second lambda shock, an expansion fan is formed at the bump crest which expands and accelerates the flow to become supersonic downstream of the bump crest. This leads to the occurrence of a favourable pressure gradient around the bump crest that suppresses the appearance of flow separation at that region. Therefore, a diffused and downwards pointing shear layer is shown at the bump crest which implies that the extent of flow separation is limited downstream of the bump crest.

Changes appear when the normal shock is impinged at the bump crest of the rounded contour bump (i.e. *l*/*c* = 0.667) as shown in Fig. 3d. A large lambda shock structure is formed at the bump crest. This lambda shock structure is composed of two barely visible front oblique shock segments/compression waves (F.F.L. and S.F.L.) as the front legs and a clearly defined normal shock rear leg (R.L.). Since the rear leg of the lambda shock (R.L.) is impinging at the bump crest; therefore, the flow immediately behind the bump crest is subsonic. This implies that the formation of the second lambda shock structure or expansion waves becomes impossible in this normal shock impingement position. In addition, a closer examination of the Schlieren image shown in Fig. 3d concluded that the shear layer is deflected upwards by the rear leg of the lambda shock. It is deduced that a strong adverse pressure gradient is presented around the rear leg of the lambda shock in the present case.

Figure 3e shows the streamwise flow pattern over the rounded contour bump when the shock impingement location is *l*/*c* = 0.833 (i.e., at the middle between the bump crest and the rear end of the bump). Two clearly defined lambda shock structures can be seen from Fig. 3e. One of the lambda shock structure has its front leg (F.F.L.) located at the front of the bump. Downstream of the front leg of the first lambda shock (F.F.L.), the supersonic flow, further expands and accelerates along the ramp-shaped front surface of the bump. As a result, the local flow Mach number in the bump crest is considerably high. Therefore, a strong oblique shock segment appears at the bump crest as the front leg of the second lambda shock structure (S.F.L.). The adverse pressure gradient induced by this considerably strong front leg of the second lambda shock (S.F.L.) causing flow separation immediately downstream where it is situated. This is evidenced by the presence of the shear layer (S.L.) at the bump crest, as shown in Fig. 3e. The rear leg of the lambda shock structure (R.L.) located downstream of the bump crest compressed and decelerated the supersonic flow downstream to become subsonic. A blurred region is shown immediately behind the rear leg of the lambda shock which indicates that the flow properties have changed across the shock.

Finally, when the normal shock impinged at the rear end of the rounded contour bump (i.e. *l*/*c* = 1) as shown in Fig. 3f, at least three lambda shock structures can be observed. Similar to all other cases being studied, the largest lambda shock structure that formed has a weak oblique shock segment/compression wave (F.F.L.) at the beginning of the bump and its rear leg is located immediately behind the rear end of the bump. However, compared with the case, when the normal shock is impinged at *l*/*c* = 0.833 (Fig. 3e), it can be seen that in the present case, when *l*/*c* = 1, the front leg of the second lambda shock structure (S.F.L.) is located at the bump valley instead of at the bump crest. Since the flow downstream of the front leg of the first lambda shock (F.F.L.) is supersonic and it expands continuously along the front surface of the bump, therefore, a considerably high flow Mach number appears at the bump crest. From Fig. 3f, it can be seen that an expansion fan (E.W.) is formed at the bump crest that further expands the flow. Flow separation also appears downstream of the bump crest which is evidenced by the presence of a shear layer (S.L.). The separated flow behind the bump crest is compressed and decelerated by the relatively strong front leg of the second lambda shock (S.F.L.). Slightly, downstream of the front leg of the second lambda shock, although less clearly defined, and the front leg of the third lambda shock structure (T.F.L.) is observed and across which the flow is further compressed and decelerated before reaching the rear end of the lambda shock (R.L.) at the rear end of the bump. It is interesting to noted that the flow features appear over the surface of the rounded contour bump surface in the present case are similar to those shown in Lo (2014), Lo and Kontis (2015, 2016) and Lo et al. (2013, 2015) using the rounded contour bump alone without normal shock impingement involved.

**Fig. 4:**
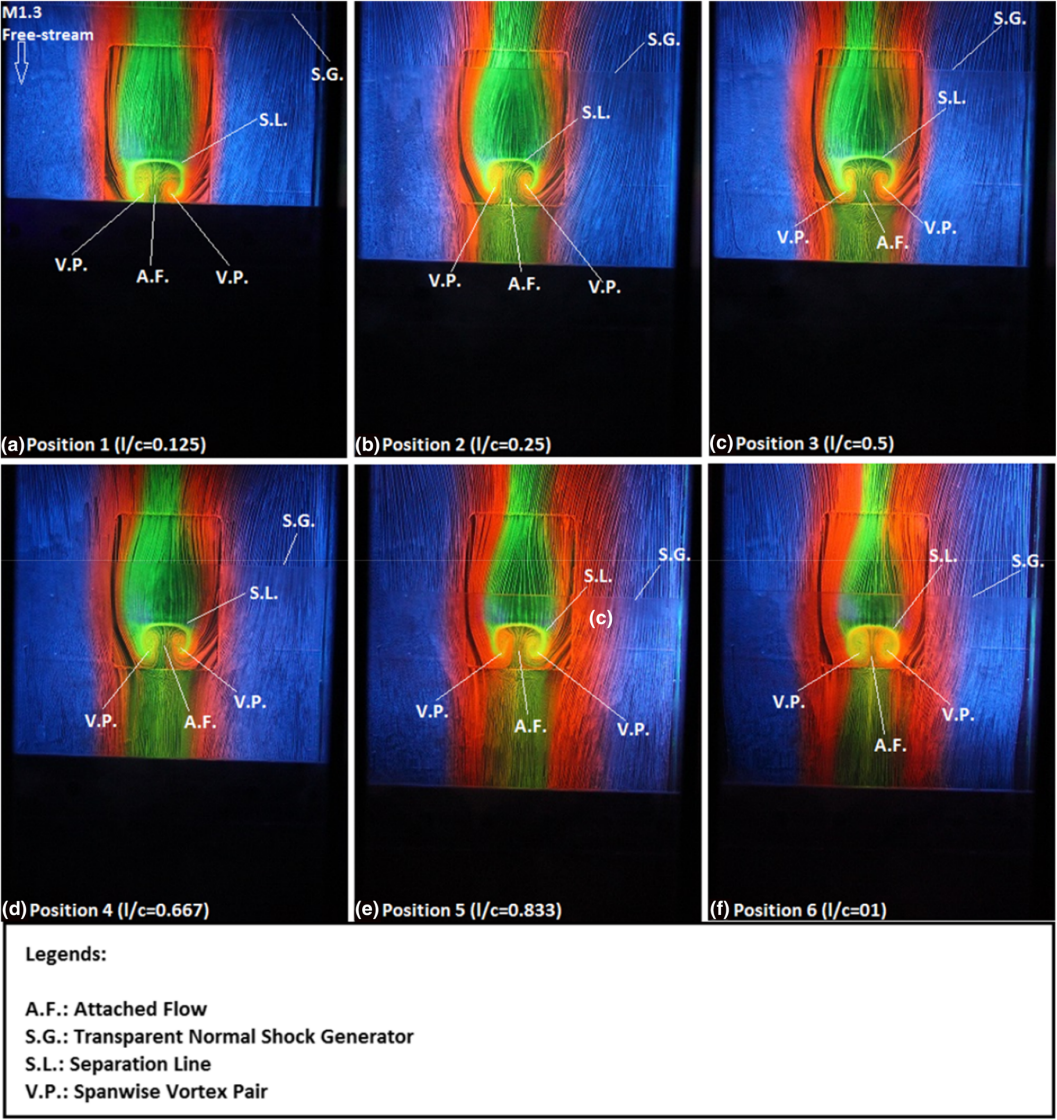
Surface oil-flow visualisation images of the nearly normal shock impinging over the surface of the rounded contour bump at various normalised (*l*/*c*) locations. **a** *l*/*c* = 0.125, **b** *l*/*c* = 0.25, **c** *l*/*c* = 0.5, **d** *l*/*c* = 0.667 (i.e., the bump crest), **e** *l*/*c* = 0.833, and **f** *l*/*c* = 1 (i.e. the rear end of the contour bump)

After considering the streamwise flow pattern, the corresponding instantaneous spanwise flow pattern is shown in Fig. 4 via a series of images captured from the surface oil-flow visualisation experiments. The advantages of using fluorescent dye with multiple colours in the surface oil-flow visualisation experiments could be clearly observed from Fig. 4. The different colour oil streaks clearly indicate the flow directions over the contour bump model. In addition, flow mixing regions in the flow field could be easily identified using this novel experimental strategy. One clear example shown in Fig. 4 is that the orange colour oil streaks present in the two spanwise vortices indicate that the flow downstream of the bump crest mixes with the flow around the bump from the two sides. This proves that using multiple colour dye in surface oil-flow visualisation experiments is beneficial in visualising complicated flow pattern that presents in the flow field.

After describing the advantages of using fluorescent oil with multiple colours in surface oil-flow visualisation experiments, the spanwise flow pattern along the rounded contour bump is discussed. When the normal shock impinging location is at *l*/*c* = 0.125 (Fig. 4a) over the surface of the rounded contour bump, the flow downstream of the rear leg of the primary lambda shock as shown in the Schlieren image (Fig. 3a) is subsonic. The flow re-expands along the ramp-shaped front surface of the contour bump model and reaches the bump crest. As mentioned earlier when discussing the result obtained from the Schlieren photography experiments, flow separation appears immediately downstream of the bump crest. This is evidenced by the presence of a separation line (S.L.) in the bump crest shown in Fig. 4a. Downstream of the bump crest, a pair of spanwise vortices (V.P.), is shown. The size of this spanwise vortex pair is small and the cores of these two vortices are not clearly defined. The formation mechanism of this spanwise vortex pair is due to the presence of a low-pressure region downstream of the bump crest attracts the flow from the two sides of the bump to move into and circulates there. This is evidenced by observing the red colour oil streaks downstream of the bump crest that the flow around the bump moves into and circulates in the bump valley. In addition, the flow from the two sides mixes with the flow that moves along the bump (i.e. the green colour oil streaks). As a result, a region that shows orange colour oil streaks is present downstream of the bump crest. In addition, a closer look into the oil streaks downstream of the bump crest reveals that the flow in the centre portion remains attached (A.F.). In fact, similar flow pattern was also reported by Ogawa et al. (2008) using a wedge-shaped bump.

Figure 4b shows the spanwise flow pattern when the normal shock impinging location is at *l*/*c* = 0.25 over the ramp-shape front surface of the bump. In this shock impinging location, the spanwise flow pattern remains similar to that shown in Fig. 4a when *l*/*c* = 0.125. The flow remains attached until it reaches the bump crest. Flow separation begins at the bump crest which is evidenced by the presence of a separation line (S.L.) there. Downstream of the bump crest, the flow remains attached in the centre portion of the bump (A.F.) and a pair of spanwise counter-rotating vortices (V.P.) is formed at the two sides. Compared with the case when the shock impinging location is at *l*/*c* = 0.125 (Fig. 4a), it can be seen that in the present case (Fig. 4b), the two spanwise vortices that formed are larger and more clearly defined. The flow pattern remains similar when the normal shock impinging location is shifted to the normalised location *l*/*c* = 0.5 (Fig. 4c), i.e., slightly upstream of the bump crest. In this case, the two spanwise counter-rotating vortices that form behind the bump crest become clearly defined, although the size becomes slightly smaller than those shown in Fig. 4b. This implies that slightly weaker flow separation occurs downstream of the bump crest.

When the normal shock impinging location is at the bump crest of the contour bump (i.e., *l*/*c* = 0.667), as shown in Fig. 4d, upstream of the bump crest, the flow pattern remains similar to those shown in Fig. 4a–c. However, downstream of the bump crest, the appearance of the flow separation at the bump crest leads to the formation of two clearly defined spanwise counter-rotating vortices (V.P.). In fact, from Fig. 4d, it can be seen that the two vortices that formed are larger than those shown when the normal shock is impinged upstream of the bump crest (Fig. 4a–c). The presence of these two clearly defined and large spanwise vortices in the present case indicates that a considerably strong flow separation occurs downstream of the bump crest. This is further evidenced by the presence of a narrower attached flow region (A.F.) adjacent to the two spanwise vortices in the present case than those shown in Fig. 4a–c. In fact, similar flow pattern was also documented in König et al. (2007, 2009), although the authors in these studies did not explain the associated flow physics about the occurrence of this phenomenon. It is deduced that as the rear leg of the lambda shock (R.L.) in the present case is located at the apex of the rounded contour bump (Fig. 3d), the impinging normal shock induces a strong adverse pressure gradient at the bump crest. This leads to the occurrence of strong flow separation at the bump crest in the present case. The spanwise flow pattern when the normal shock is impinged at *l*/*c* = 0.833 downstream of the bump crest shown in Fig. 4e remains similar to that shown in Fig. 4d when the normal shock is impinged at the bump crest. One observable difference between these two cases is that the spanwise counter-rotating vortex pair (V.P.) is even larger and their vortex cores are more clearly defined in the present case when the normal shock impinging location is at *l*/*c* = 0.833.

Finally, Fig. 4f shows the spanwise flow pattern when the normal shock is impinged immediately downstream of the rear end of the rounded contour bump. The flow features remain similar to those shown in Fig. 4a–e when the normal shock is impinged over the bump surface at more upstream locations. However, in this case, two large spanwise counter-rotating vortices (V.P.) are presented behind the bump crest which almost covered the entire bump valley region. In addition, as shown in Fig. 4f, the attached flow region (A.F.) downstream of the bump crest adjacent to the two spanwise vortices becomes extremely narrowed and can only be barely seen. This implies that the extent of flow separation that occurs in the present case is the strongest amongst all other cases being studied. In fact, the flow features shown in Fig. 4f are similar to those shown in Lo (2014), Lo and Kontis (2016) and Lo et al. (2013, 2015) when a rounded contour bump alone is subjected to a supersonic free-stream. This is reasonable as in the present case, the rear leg of the lambda shock (R.L.) is located behind the rear end of the bump, as shown in the Schlieren image (Fig. 3f). Therefore, the flow pattern over the contour bump upstream of the rear leg of the lambda shock should be similar to the flow pattern over the rounded contour bump without normal shock impingement involved.

### Wind-on versus wind-off spanwise flow pattern

**Fig. 5:**
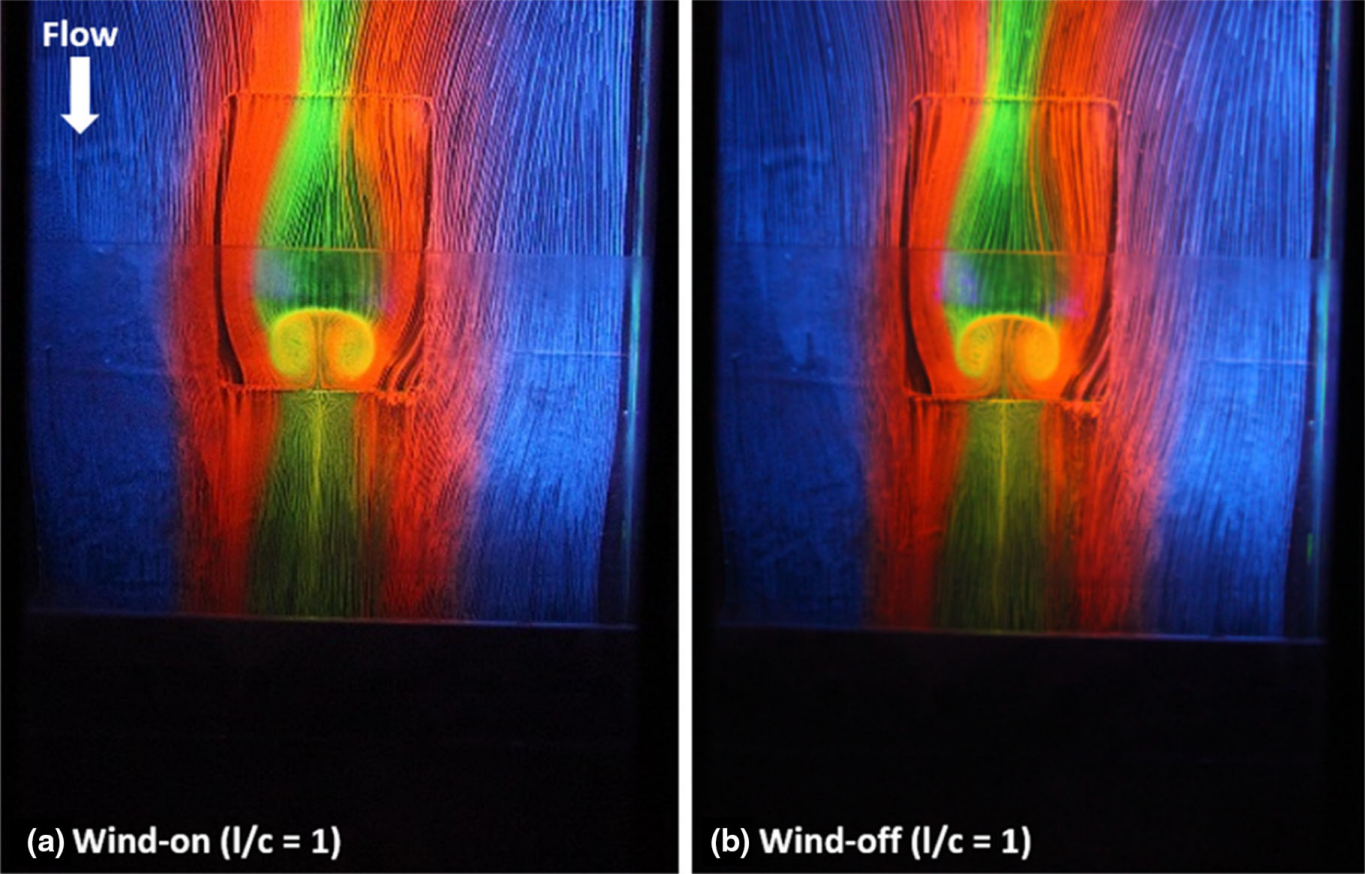
Images taken from the surface oil-flow visualisation experiment. **a** Wind-on and **b** wind-off images

As the major objective of this study is to investigate the advantages and disadvantages of using the transparent shock generator in experiments which involved normal shock impingement. The advantages of using the transparent shock generator to capture the instantaneous spanwise flow pattern in the wind-on condition are well illustrated in Fig. 5. Figure 5a, b are the images captured from the surface oil-flow visualisation experiment in the wind-on and wind-off condition, respectively. In general, it can be seen from these two images that the oil streaks left on the surface of the contour bump model are similar in both cases. However, a closer look into these images can reveal that the image captured in the wind-off condition (Fig. 5b) is more blurred compared with the instantaneous oil streaks captured in the wind-on condition (Fig. 5a). It should be noted that the two images shown in Fig. 5 were captured in the same set of experiment and the camera settings remained unchanged throughout the entire period of experiment. Therefore, the blurred image shown in Fig. 5b was solely caused by the effects of the backward moving terminating shock in the shutdown period of the supersonic wind tunnel used in the experiment.

In addition to the more clearly defined wind-on image, it can be clearly seen from Fig. 5a that the two spanwise vortices captured in the wind-on condition are considerably larger and more symmetrical. Moreover, their vortex cores are clearly defined and the flow mixing phenomenon within these vortices can be easily observed. In contrast, due to the presence of the backward moving terminating shock in the wind tunnel shut down period, these spanwise vortices shown in the image captured in the wind-off condition (Fig. 5b) are smaller and more distorted. It is particularly clear when the spanwise vortex in the left side shown in Fig. 5b is considered. In fact, the oil streaks shown in Fig. 5b suggest that the left spanwise vortex is located more upstream (i.e., closer to the bump crest) than the vortex at the right-hand-side with its top portion merged with the separation line. This wind-off spanwise flow pattern captured is completely different from that shown in the wind-on image (Fig. 5a). It should be noted that the images shown in Fig. 5 have been scaled down from two much higher resolution images. Therefore, the difference in the details of the flow features that shown in the raw images is more significant and much easier to be identified.

Based on the results shown in Fig. 5, it is clear that the spanwise flow pattern shown in the wind-on and wind-off conditions is different. The instantaneous flow pattern captured in the wind-on condition is always more clearly defined than that captured in the wind-off condition. The result shown in Fig. 5 emphasises the importance in capturing instantaneous flow pattern in the wind-on condition in the experimental study. It also demonstrates the advantage of using the transparent shock generator in the present experimental study that involves the use of the transonic/supersonic wind tunnel. In addition, as previously mentioned, the transparent nature of the shock generator means that some other experimental techniques, such as instantaneous particle image velocimetry, particle shadow velocimetry, and liquid crystal flow visualisation and measurements, as well as potentially infra-red thermography could be conducted. With the state-of-the-art addictive manufacturing techniques, it is foreseeable that lighter, sharper, and tougher transparent shock generators could be manufactured and would be more commonly used in experimental studies in the coming future.

## Conclusion

An experimental study has been conducted to investigate the problem of normal shock impinging over a rounded contour bump in a Mach 1.3 free-stream using a transonic/supersonic wind tunnel. A piece of novel transparent normal shock wave generator was used to generate the impinging normal shock wave. Multiple colour surface oil-flow visualisation and monochrome high-speed Schlieren photography techniques were used for flow diagnostics. The flow pattern of the rounded contour bump was examined over six normalised shock wave impinging locations ranging from 0.125 < *l*/*c* < 1. The result obtained from the Schlieren photography experiments shows that the rounded contour bump could split the impinging normal shock into a or a series of weaker lambda shock structures. It was observed that in addition to the primary lambda shock, additional independent lambda shock structures were formed in the bump crest region when the normal shock is impinged over the ramp-shape front surface of the rounded contour bump. In contrast, no independent second lambda shock structure and expansion waves could be observed when the location of the normal shock impingement was located at the apex of the rounded contour bump. However, the shear layer that formed immediately downstream of the normal shock impingement location was deflected upwards by the rear leg of the primary lambda shock structure. It is deduced that a strong adverse pressure gradient is presented in the apex region of the rounded contour bump in this normal shock impingement location. In addition, it was observed that when the location of normal shock impingement is located behind the rear end of the rounded contour bump, the resulting flow pattern that was observed from the Schlieren images becomes similar to that when a Mach 1.3 free-stream flow over a rounded contour bump without any normal shock impingement involved. In general, the result obtained from the Schlieren photography experiments was comparable with the data shown in several similar studies documented in literature.

Based on the result obtained from the surface oil-flow visualisation experiments, it can be concluded that the flow pattern over the surface of the rounded contour bump remains similar regardless of the location of the normal shock impingement. The presence of the separation line at the apex of the rounded contour bump indicates that flow separation appears downstream of the bump crest. The occurrence of flow separation leads to the formation of two counter-rotating spanwise vortices in the valley of the rounded contour bump. It was observed that the size of these spanwise vortices increased progressively when the normal shock impinging location was shifted downstream. Similarly, it was found that a region which shows attached flow appeared in the centre portion of the bump valley, while its size decreased progressively when the normal shock impinging location was moved downstream. Moreover, it was concluded that the spanwise flow features that formed over the bump surface when the normal shock impingement location was at *l*/*c* = 1 became similar to those obtained when the rounded contour bump is subjected to a Mach 1.3 free-stream without any shock impingement involved. The advantages of using multiple colour dye in the surface oil-flow experiments have clearly been demonstrated. The three colours fluorescent oil used generally enhanced the visualisation of the complicated flow features and flow mixing regions that presented in the flow field.

Finally, the flow pattern captured in the surface oil-flow visualisation experiment in the wind-on and wind-off condition was compared. It was observed that images captured instantaneously in the wind-on condition are more clearly defined. In addition, the distortion of flow features was identified in the oil streaks captured in the wind-off condition. The advantages and potential shortfalls of using the transparent normal shock wave generator have been discussed.
